# Reply to Comment on Pietrapertosa et al. How to Prioritize Energy Efficiency Intervention in Municipal Public Buildings to Decrease CO_2_ Emissions? A Case Study from Italy. *Int. J. Environ. Res. Public Health* **2020**, *17*, 4434

**DOI:** 10.3390/ijerph18073760

**Published:** 2021-04-04

**Authors:** Filomena Pietrapertosa, Marco Tancredi, Michele Giordano, Carmelina Cosmi, Monica Salvia

**Affiliations:** 1 Institute of Methodologies for Environmental Analysis, National Research Council of Italy (CNR-IMAA) C.da S. Loja, 85050 Tito Scalo PZ, Italy; carmelina.cosmi@imaa.cnr.it (C.C.); monica.salvia@imaa.cnr.it (M.S.); 2 Lucanian Energy Company, SEL. Corso Umberto I, 28, 85100 Potenza PZ, Italy; marcotanc@gmail.com (M.T.); michele.giordano@selspa.it (M.G.)

This is a reply to the paper by Miroslav Variny [1] that commented on our recently published work, “How to prioritize energy efficiency intervention in municipal public buildings to decrease CO_2_ emissions? A case study from Italy” [2].

The authors greatly appreciate the careful review carried out by Dr. Variny and his useful comments, which will be taken into serious consideration for further studies aimed at improving the presented Decision Support Tool (DSTool) and expanding its future applications.

While finding relevant the observations made by Dr. Variny, we would like to underline that the DSTool is not suitable to be used as an individual approach to investigate single buildings in detail. In fact, it was designed and implemented with the broad aim of providing decision-makers and technicians with an easy-to-use tool to compare a portfolio of energy efficiency (EE) and renewable energy (RES) interventions within the stock of municipal public buildings (MPB) and provide an evaluation of the investment opportunities in terms of costs, energy savings and avoided CO_2_ emissions [3]. In fact, nZEB (Nearly Zero Energy Building) conditions, the overarching target and the gold standard, are difficult to achieve without carrying out an in-depth renovation of the existing buildings, within the limited available budget of public administrations.

To increase the energy performance of public buildings and pave the way towards the achievement of nZEB standards, considering the physical and economic constraints, cost-effective interventions should be identified comparing their costs, energy savings, return on investment (ROI) and CO_2_ emission reduction.

Within the framework of the PrioritEE project, the aim was to design and implement a flexible instrument, the DSTool, that could effectively support decision-making by providing preliminary indications about the priority of the interventions that can increase, on the whole, the energy and environmental performance of public buildings within the available budget and the ongoing renovation plans, by selecting the buildings in which it is more convenient to invest and the most suitable interventions.

The DSTool can be adapted to different user needs, climates, energy use profiles, building typologies and regulations to take into account the variety of the five considered pilot regions. Two levels of input data, basic and advanced, are used to characterise the building stock, considering either the organisational capacities of involved staff or the level of detail of the available information. The “Calculations” section estimates a set of indicators for the current building status to be used as a benchmark, while the “Results” section presents all necessary information for ranking and prioritising EE and RES interventions in each MPB and in the whole stock of MPBs considered [4]. The DSTool was widely tested in five pilot case studies, giving comparable results, and the small differences observed were mainly due to the limits of the input data [5]. In the following, some clarifications on the main remarks are provided.

There is a noticeable uncertainty in estimating CO_2_ emissions and, as observed by Miroslav Variny, average electricity emission factors “differ for each country based on its energy mix”. The reference document for CO_2_ emission calculation for the signatories of the Covenant of Mayors is represented by the Reporting Guidelines of the Covenant of Mayors for Climate and Energy [6]. These guidelines “strongly recommend replacing the default emission factors with country-specific emission”. The amounts of CO_2_ emissions and CO_2_ emissions saved were thus estimated by utilising the country-specific emission factors of the IEA [7] for the year 2016, a milestone for consumption data (2.2 ton CO_2_/toe, corresponding to 411.44 g CO_2_/kWh, taking into account the conversion value set by the National Authority for Electricity and Gas: 1 toe = 5347 kWh) [8].

The methodology focused on the final energy consumption rather than the reduction of the primary energy required, to subsequently highlight the contribution of behavioural changes to the reduction of energy demand [9].

More information on the technical options included in the DSTool and applied in the case studies, is described in detail in the *Technology Analytical Database*, a major component of the PrioritEE toolbox [10]. It consists of a compilation of technological solutions to improve energy efficiency in MPBs according to end-use: lighting, space heating, space cooling, water heating and cooking.

In the Potenza case study, we selected only the technologies that could be activated in a three-year action plan aimed at improving the energy performance of public buildings and increasing the use of renewable sources within a limited budget.

To conclude, the authors would like to underline that the purpose of the paper was to illustrate the application of the DSTool for the definition of the three-year action plan for the Municipality of Potenza, identifying no-regret interventions that allow the improvement of public building performance at low cost.
